# The relationship between depression and overactive bladder/urinary incontinence symptoms in the clinical OAB population

**DOI:** 10.1186/s12894-016-0179-x

**Published:** 2016-10-06

**Authors:** H. Henry Lai, Baixin Shen, Amar Rawal, Joel Vetter

**Affiliations:** 1 Department of Surgery, Division of Urologic Surgery, Washington University School of Medicine, 4960 Children’s Place, Campus Box 8242, St Louis, MO 63110 USA; 2 Department of Anesthesiology, Washington University School of Medicine, 4960 Children’s Place, Campus Box 8242, St Louis, MO 63110 USA

**Keywords:** Overactive bladder, Urinary incontinence, Urinary urgency, Depression, Psychosocial

## Abstract

**Background:**

To investigate the relationship between depression and overactive bladder (OAB)/urinary incontinence symptoms among the clinical OAB population.

**Methods:**

Patients who were diagnosed with overactive bladder (OAB) and age-matched control subjects without OAB were enrolled. Depression symptoms were assessed using the Hospital Anxiety and Depression Scale (HADS-D). OAB/incontinence symptoms were assessed using the validated questionnaires: ICIQ-UI, ICIQ-OAB, UDI-6, IIQ-7, and OAB-q.

**Results:**

27.5 % of OAB patients in our study had depression (HADS ≥8), and 12 % of OAB patients had moderate to severe depression (HADS-D ≥11). OAB patients reported significantly higher HADS-D depression scores compared to age-matched controls (5.3 ± 3.9 versus 2.8 ± 3.9, *p* = 0.004). OAB patients with depression reported more severe incontinence symptoms (ICIQ-UI), greater bother and more impact on quality of life (UDI-6, IIQ-7) compared to OAB patients without depression (*p* = 0.001, 0.01, <0.001, respectively). However there were no differences in ICIQ-OAB and OAB-q. Among OAB patients, there were positive correlations between the severity of depression symptoms and OAB/incontinence symptoms (*p*-values <0.001 to 0.035).

**Conclusions:**

27.5 % of OAB patients have depression. OAB patients with depression reported more severe urinary incontinence symptoms, greater bother and more impact on quality of life compared to those without depression. Future studies are needed to further examine the mechanistic links between depression and OAB/urinary incontinence.

## Background

Overactive bladder (OAB) affects up to 1 in 6 adult men and women in the United States [1]. The syndrome is characterized by urinary urgency, with or without urgency incontinence, usually with frequency and nocturia, in the absence of infection or other identifiable causes [2]. Given the symptom bother and impact on quality of life, it is anticipated that many OAB patients would have psychosocial difficulties. A recent review suggested that depression might be associated with OAB [3]. However most of the published studies were population-based epidemiological surveys. Surprisingly there was been very few papers that focused on OAB patients who presented to clinics [4–8].

Chiara et al. [6] compared the depression scores between female patients with stress incontinence, urgency incontinence, and mixed incontinence, and showed no differences in the depression scores between the three groups. In contrast, Stach-Lempinen et al. [7] showed that the odds of depression was significantly higher in female patients with urgency incontinence compared to those with stress incontinence (OR 3.7, 95 % CI 1.30-10.49, *p* = 0.026). Melville et al. [4] also showed that the odds of depression was higher in female patients with urgency incontinence or mixed incontinence compared to stress incontinence (OR 9.2-11.5). None of the studies above recruited a control group. In the only paper that recruited a control group, Zorn et al. [8] showed that patients with idiopathic urgency incontinence reported higher depression scores than controls who did not have incontinence. Overall the results of the studies were inconclusive.

A few studies have compared urinary incontinence (UI) patients with depression versus UI patients without depression. Melville et al. [4] showed that there was no difference in the daily UI episodes or the percent with moderate/large UI between UI patients with depression versus those without depression. Sung et al. [5] studied female obese UI patients, and also showed that there was no difference in the numbers of urgency incontinence episodes between obese UI patients with depression versus obese UI patients without depression. However, two other studies have reported higher Urogenital Distress Inventory (UDI) scores among UI patients with depression [4, 5].

It is evident that the few publications that have studied the clinical UI population yielded conflicting results [4–8]. All of the studies have recruited patients with a myriad of UI symptoms (stress, urgency, and mixed incontinence) or causes (idiopathic, obstructive, and neurogenic incontinence), thus the relationship between depression and OAB may be confounded. Most studies have examined urinary incontinence (UI) in general but have not focused on OAB specifically. To our knowledge none of the studies have specifically recruited OAB patients, compared OAB patients with versus without depression, and have correlated the severity of their depression and OAB symptoms.

Here we address the gap in the literature by specifically investigating the relationship between depression and OAB in the clinical OAB population. We have: (1) recruited OAB patients and age-matched controls, (2) compared their depression symptoms, (3) compared OAB patients with versus without depression, and (4) performed correlation analyses between the severity of their depression and OAB/incontinence symptoms.

## Methods

### Subjects

Between October 2012 and July 2014, adult patients, aged 18 or above, diagnosed with OAB and age-matched controls without OAB were recruited into this study that inquired their depression and urinary symptoms. For OAB, patients must complain of urinary urgency, with or without urgency incontinence, usually with frequency and nocturia, in the absence of infection or other identifiable causes, in accordance with the 2002 ICS (International Continence Society) definition of OAB [2]. The clinical evaluation was performed by one clinician (HL) and followed the published AUA guidelines [9]. Subjects with a history of urinary incontinence surgery, prostate surgery, urethral stricture disease, neurogenic bladder, urinary retention, pelvic radiation, tuberculosis cystitis, cyclophosphamide cystitis, genitourinary cancer, urinary stones, a documented positive urine culture in the past 6 weeks, or a post-void residual volume ≥150 mL were not eligible. Controls were recruited by local advertisement and research database. Controls must have no prior diagnosis of OAB or interstitial cystitis/bladder pain syndrome, no significant lower urinary tract symptoms (AUA symptom index < 7), no bladder or pelvic pain, and no evidence of urinary infection. Controls were age-matched to the OAB cohort. Age matching was operationalized by recruiting similar percentages of patients and controls in the following age bins: <35, 35–49, and ≥50 years old. All subjects signed an informed consent. The Washington University School of Medicine Institutional Review Board approved this study.

### Assessment

Depression symptoms were assessed using the Hospital Anxiety and Depression Scale (HADS) [10]. The questionnaire contains 7 items to assess depression symptoms (HADS-D) and 7 items to assess anxiety symptoms (HADS-A). A score of ≥8 on the HADS-D was used to operationalize the presence of depression [11]. We have also used a cut-offs of HADS-D 8–10 to indicate mild depression and ≥11 for moderate/severe depression. The developers of the HADS have recommended these cut-off points for mild (8–10), moderate (11–14), and severe (15–21) depression [10, 11]. To quantify the severity of depression symptoms, we have analyzed the HADS-D data as a continuous variable.

OAB/incontinence symptoms were assessed using the following validated questionnaires: 1) International Consultation on Incontinence – Urinary Incontinence Short Form (ICIQ-UI) [12], 2) International Consultation on Incontinence – Overactive Bladder (ICIQ-OAB) [13], 3) OAB-q Short Form [14], 4) Urogenital Distress Inventory Short Form (UDI-6) [15], and 5) Incontinence Impact Questionnaire Short Form (IIQ-7) [15]. Briefly, ICIQ-UI is a 4-item questionnaire that assesses the frequency, amount and interference of urinary incontinence. ICIQ-OAB is a 4-item questionnaire that inquires about daytime frequency, nighttime frequency, urgency, and urgency incontinence. OAB-q contains two sub-scales that assess symptom bother and condition-specific quality of life. UDI-6 and IIQ-7 measure urinary distress and incontinence impact.

### Statistical analysis

Linear regression models (continuous variables) were used for multivariate comparisons between OAB and controls, and between the OAB subgroups (with versus without depression), adjusting for age (continuous variable) and sex. Other potential covariates were not included in the models due to the given sample size. Spearman’s correlation was used for correlation analyses. *p* < 0.05 was considered significant difference. Data were presented as mean ± SD in the tables. All statistical analysis was completed using the open source statistical package R v3.2.0.

## Results

### Study population

Fifty-one adult OAB patients and 30 age-matched controls participated in this study. Appendix 1 described the study population (demographics, urinary symptoms, validated questionnaires, and medical comorbidities). The mean age (± SD) of the OAB and the control groups was 53.8 ± 11.9 and 54.2 ± 12.3, respectively. There was no significant age or sex difference between the two groups (*p* = 0.984 and 0.14). As expected, OAB patients had worse OAB/incontinence symptoms and quality of life compared to controls.

### Depression symptoms among OAB patients, and comparison to controls

OAB patients reported significantly higher depression scores than controls on the HADS-D (5.3 ± 3.9 versus 2.8 ± 3.9, *p* = 0.004, Table 1). The HADS-D scores were almost 90 % higher in OAB patients compared to controls. Looking at the individual items on the HADS-D scale, OAB patients fared worse compared to controls on the following items: “I still enjoy the things I used to enjoy;” “I can laugh and see the funny side of things;” “I feel as if I am slowed down;” and “I look forward to enjoyment to things.” (*p* = 0.044, 0.025, <0.001, 0.03, respectively). 27.5 % of OAB patients reported a HADS-D score ≥8, a threshold commonly used to indicate clinically relevant depression symptoms. 11.8 % OAB patients reported a HADS-D score ≥11, indicating moderate to severe depression symptoms. 32 % of OAB patients reported a medical history of depression.

**Table 1:** Comparison of depression symptoms between OAB and controls (adjusted for age and sex), *p < 0.05

	OAB patients (*n* = 51)	Controls (*n* = 29)^a^	*p*-value (adjusted for age and sex)
HADS-D composite scores: (mean ± SD)	5.3 ± 3.9	2.8 ± 3.9	0.004*
Individual items on the HADS-D scale: (higher score is worse)			
I still enjoy the things I used to enjoy:	0.88 ± 0.86	0.47 ± 0.90	0.044*
0 = definite as much			
1 = not quite so much			
2 = only a little			
3 = hardly at all			
I can laugh and see the funny side of things:	0.45 ± 0.58	0.17 ± 0.46	0.025*
0 = as much as I always could			
1 = not quite so much now			
2 = definite not so much now			
3 = not at all			
I feel cheerful:	0.59 ± 0.64	0.43 ± 0.73	0.21
3 = not at all			
2 = not often			
1 = sometimes			
0 = most of the time			
I feel as if I am slowed down:	1.4 ± 0.90	0.55 ± 0.57	<0.001*
3 = nearly all the time			
2 = very often			
1 = sometimes			
0 = not at all			
I have lost interest in my appearance:	0.76 ± 0.91	0.57 ± 0.86	0.16
3 = definitely			
2 = I don’t take as much care as I should			
1 = I may not take quite as much care			
0 = I take just as much care as ever			
I look forward with enjoyment to things:	0.75 ± 087	0.33 ± 0.66	0.03*
0 = as much as I ever did			
1 = rather less than I used to			
2 = definitely less than I used to			
3 = hardly at all			
I can enjoy a good book or radio or Tv program:	0.41 ± 0.67	0.23 ± 0.63	0.22
0 = often			
1 = sometimes			
2 = not often			
3 = very seldom			
% with a HADS-D score ≥8 (presence of depression):	27.5 %	13.8 %	0.158
% with a HADS-D score ≥11 (moderate to severe depression):	11.8 %	10.3 %	0.744
% with medical history of depression:	32 %	17 %	0.196

^a^One control did not provide complete HADS-D data and was thus excluded from comparisons

### Comparison of OAB patients with versus without depression symptoms

**Fig. 1:**
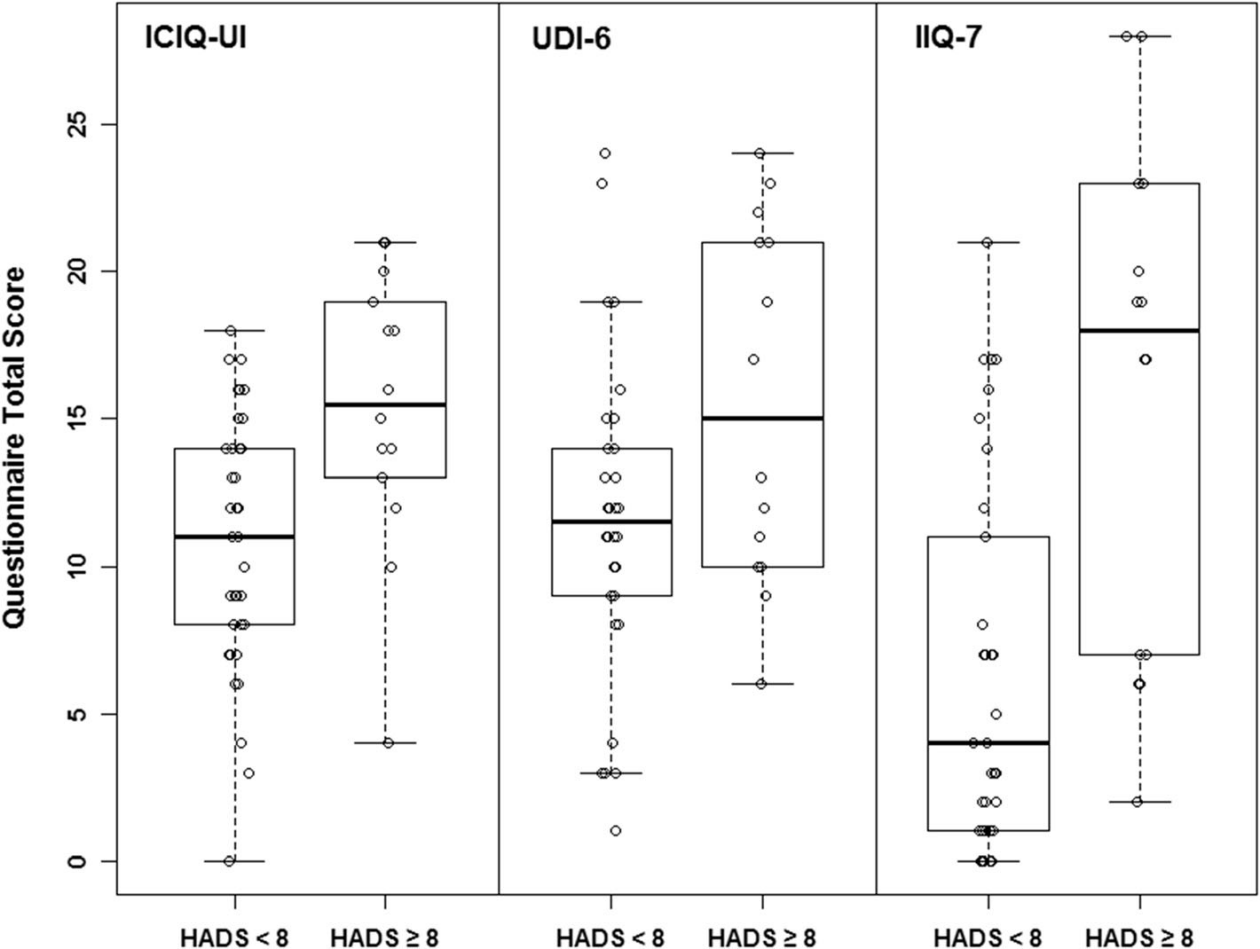
OAB patients with anxiety had higher incontinence scoresᅟ

The OAB patients were categorized into two subgroups: those with depression (HADS-D score ≥8, 27.5 %) versus those without depression (HADS-D <8, 72.5 %). Their OAB/incontinence symptoms and quality of life were then compared between the two OAB subgroups. As shown in Table 2, OAB patients with depression reported more severe incontinence symptoms (ICIQ-UI), greater bother and more impact on quality of life (UDI-6, IIQ-7) compared to OAB patients without depression (*p* = 0.001, 0.01, <0.001, respectively). However there were no differences in ICIQ-OAB and OAB-q. The box plots of their ICIQ-UI, UDI-6 and IIQ-7 scores were presented in Fig. 1.

**Table 2:** Comparison of OAB/incontinence symptoms and quality of life between OAB patients with depression versus without depression (adjusted for age and sex), *p < 0.05

	OAB patients with depression (HADS-D ≥8)	OAB patients without depression (HADS-D <8)	*p*-value (adjusted for age and sex)
Demographics:			
No. of subjects	14 (27.5 %)	37 (72.5 %)	
Age (mean ± SD)	56.2 ± 9.9	52.8 ± 12.6	0.44
Sex (% females)	64 %	76 %	0.42
HADS-D scores	10.4 ± 2.4	3.3 ± 2.2	<0.001*
Validated questionnaires: (mean ± SD)			
ICIQ-UI (urinary incontinence, 0-21)	15.4 ± 4.7	10.8 ± 4.3	0.001*
UDI-6 (Urogenital Distress Inventory, 0-24)	15.6 ± 6.1	11.5 ± 5.1	0.010*
IIQ-7 (Incontinence Impact Questionnaire, 0-28)	15.9 ± 8.7	6.1 ± 6.2	<0.001*
ICIQ-OAB (overactive bladder, 0-16)	10.1 ± 3.2	9.0 ± 2.4	0.12
OAB-q symptom bother subscale (6-36)	19.7 ± 7.9	18.9 ± 6.1	0.51
OAB-q quality of life subscale (13-78)	36.6 ± 19.1	26.7 ± 15.2	0.074

*Statistically significant factors using multivariate logistic regression model

Since most of the validated urinary questionnaires assess multiple urinary symptoms and presented the results as composite scores, we performed an exploratory analysis to compare the difference in individual urinary symptoms between OAB patients with and without depression (Table 3). The results suggested that that depression was correlated with urinary incontinence or urgency incontinence symptoms (ICIQ-UI questions 3, 4, and ICIQ-OAB question 6a). However, the differences in urgency, frequency, and nocturia symptoms did not meet criteria for significance (*p >* 0.05).

**Table 3:** Comparison of individual urinary symptoms (adjusted for age and sex), *p < 0.05

	OAB patients with depression (HADS-D ≥8), *n* = 14	OAB patients without depression (HADS-D <8), *n* = 37	*p*-value (adjusted for age and sex)	Source of question
Incontinence symptoms:				
How often do you leak urine?^a^	4.1 ± 1.1	2.8 ± 1.3	0.002*	ICIQ-UI question 3
How much urine do you usually leak?^b^	3.7 ± 1.7	2.4 ± 1.2	0.002*	ICIQ-UI question 4
Does urine leak before you can get to the toilet?^c^ (urgency incontinence)	2.8 ± 0.9	2.0 ± 0.8	0.003*	ICIQ-OAB question 6a
Frequency or nocturia symptoms:				
How many times do you urinate during the day? (see footnote)^d^	1.7 ± 1.3	1.8 ± 1.2	0.98	ICIQ-OAB question 3a
During the night, how many times do you have to get up to urinate, on average?^e^	2.6 ± 1.2	2.6 ± 1.1	0.93	ICIQ-OAB question 4a
Numeric rating scale of frequency (0-10)	6.4 ± 3.3	6.4 ± 2.3	0.86	0-10 numeric rating scale
Urgency symptoms:				
Do you have to rush to the toilet to urinate?^f^	2.9 ± 0.8	2.6 ± 0.9	0.10	ICIQ-OAB question 5a
Numeric rating scale of urgency (0-10)	6.9 ± 2.9	5.9 ± 2.4	0.18	0-10 numeric rating scale
USS (urgency severity scale, 0-3)^g^	2.1 ± 0.9	2.1 ± 0.7	0.83	USS is a 4-point self-reported rating scale of the degree of urgency sensation (none, mild, moderate, severe).^g^

*Statistically significant factors using multivariate logistic regression model

Based on the categories of response on the ICIQ questionnaire

^a^How often do you leak urine? 0 = never, 1 = about once a week or less often, 2 = two or three times a week, 3 = about once a day, 4 = several times a day, 5 = all the time

^b^How much urine do you usually leak? 0 = none, 2 = a small amount, 4 = a moderate amount, 6 = a large amount

^c^Does urine leak before you can get to the toilet? 0 = never, 1 = occasionally, 2 = sometimes, 3 = most of the time, 4 = all of the time

^d^How many times do you urinate during the day? 0 = 1 to 6 times, 1 = 7 to 8 times 2 = 9 to 10 times, 3 = 11 to 12 times, 4 = 13 or more times

^e^During the night, how many times do you have to get up to urinate, on average? 0 = none, 1 = one time, 2 = two times, 3 = three times, 4 = four or more times

^f^Do you have to rush to the toilet to urinate? 0 = never, 1 = occasionally, 2 = sometimes, 3 = most of the time, 4 = all of the time

^g^Urgency severity scale (USS) reference: Nixon, A., Colman, S., Sabounjian, L. et al.: A validated patient reported measure of urinary urgency severity in overactive bladder for use in clinical trials. J Urol, 174: 604, 2005

### Correlation between the severity of depression symptoms and OAB/incontinence symptoms

To quantify the severity of depression symptoms, we have analyzed the HADS-D data as a continuous variable (0 to 21). We then performed Spearman’s correlation analyses between HADS-D and the urinary questionnaires (Table 4). Among OAB patients, there were positive correlations between the severity of depression symptoms (HADS-D) and the severity of OAB/incontinence symptoms, symptom bother and impact on quality of life (ICIQ-UI, ICIQ-OAB, UDI-6, IIQ-7, OAB-q, *p*-values <0.001 to 0.035). The highest correlations were between HADS-D and IIQ-7, and between HADS-D and OAB-q quality of life (correlation coefficient = 0.53 and 0.40 respectively, *p*-values <0.001 and = 0.001 respectively).

**Table 4:** Spearman’s correlation analyses between the severity of depression symptoms (HADS-D from 0 to 21) and OAB/incontinence symptoms among OAB patients (*n* = 51), *p < 0.05

	Spearman’s correlation coefficient (to HADS-D)	*p*-value (multivariate linear regression, adjusted for age and sex)
Urinary questionnaires:		
ICIQ-UI (urinary incontinence, 0-21)	0.29	0.006*
UDI-6 (urogenital distress inventory, 0-24)	0.32	<0.001*
IIQ-7 (incontinence impact questionnaire, 0-28)	0.53	<0.001*
ICIQ-OAB (overactive bladder, 0-16)	0.17	0.023*
OAB-q symptom bother subscale (6-36)	0.21	0.035*
OAB-q quality of life subscale (13-78)	0.40	0.001*

*Statistically significant factors using multivariate logistic regression model

## Discussion

The present study specifically recruited OAB patients. We compared the depression symptoms between OAB and controls, and the urinary symptoms between the two OAB subgroups – with and without depression. The results showed that: (1) OAB patients have higher depression scores than controls; (2) OAB patients with depression reported more severe incontinence, greater bother and more impact on quality of life than OAB patients without depression; (3) there are positive correlations between the severity of depression symptoms and OAB/incontinence symptoms among the OAB patients.

27.5 % of OAB patients in our study have depression symptoms based on the HADS-D scale (HADS-D ≥8). Moreover, more than 10 % of OAB patients in our study have moderate to severe depression (HADS-D ≥11). The high prevalence of depression in the clinical OAB population has therapeutic implications. Behaviors associated with depression may impede response to clinical intervention. For example, behavioral therapies for OAB (e.g., bladder training, dietary and fluid modification, pelvic floor muscle exercises) require active participation of the patient. Depressed patients may lack the self-motivation, desire, and persistent effort necessary to have a successful outcome with behavioral therapies. Depression may also influence compliance to pharmacological therapies. Since the goals of OAB treatment are not only to improve symptoms but also to improve quality of life and function, not recognizing depression and its impact may contribute to suboptimal improvement in patient’s global impression of improvement (PGI-I). Urologists or urogynecologists are usually not trained to diagnose and treat clinical depression. However, they can screen OAB patients, particularly those with severe urinary incontinence, for depressive symptoms using the HADS-D scale. HADS-D takes less than a minute for patients to complete. OAB patients that are flagged for depressive symptoms might be considered for referral. It is important to recognize psychosocial comorbidities in patients seeking care because of their potential influence on therapeutics and high prevalence (~30 % of OAB patients have depression, and >10 % have moderate to severe depression).

We have demonstrated the positive associations between the severity of depression symptoms and OAB/incontinence symptoms in this cross-sectional study. However, to the best of our knowledge, the nature behind this association is unknown. It is unclear which came first, the “chicken” or the “egg.” While it is not hard to imagine that the symptoms and functional impairment associated with OAB/incontinence may lead to depression, the possibility that OAB and depression may share a common pathophysiological pathway in some patients deserves further research. Selective serotonin uptake inhibitors are commonly used to manage depression. Reduction of serotonin levels in the central nervous system is associated with increased urinary frequency and bladder contractions, while activation of the central serotonergic system with a 5-HT uptake inhibitor depresses bladder contractions and increases the micturition threshold volume in animal studies [16]. Thus depression may be mechanistically linked to OAB via the serotonergic pathway. In an earlier clinical study, duloxetine – a serotonin noradrenergic uptake inhibitor – has been shown to improve both the “wet” and “dry” symptoms in OAB patients [17].

A few population-based cohort studies have tried to clarify the relationship between depression and OAB. In an one-year longitudinal study of women aged 40 years and over, incident cases of depression was predicted by the presence of urgency incontinence at baseline; while incident cases of urgency incontinence was not predicted by depression at baseline [18]. In two longitudinal studies involving older patients aged 65 and above, depression at baseline predicted new onset urgency incontinence at the 1-year follow up in one study and at the 6-year follow up in the second study [19, 20]. Studies in the clinical OAB population showed that depression improved with successful treatment of OAB with anticholinergic medications, botulinum toxin A, and InterStim neuromodulation [21–23]. Collectively these studies suggested a causality relationship between depression and OAB/incontinence. Future studies are needed to further examine the mechanistic links between depression and OAB.

It is interesting to note that in Table 2, we demonstrated differences in ICIQ-UI, UDI-6 and IIQ-7 but not in ICIQ-OAB or OAB-q between OAB patients with and without depression. As shown in Table 3, the main difference between OAB patients with and without depression was due to their incontinence rather than their urgency or frequency symptoms. Thus questionnaires that focus more on the incontinence symptoms (ICIQ-UI, UDI-6 or IIQ-7) captured the differences while questionnaires that focus more on urgency/frequency symptoms (OAB-q or ICIQ-OAB) may not have captured a difference.

The current study has limitations: 1) It was a single-institution study with small sample size, thus it may not have sufficient power for more detailed analyses (e.g., adjusting for additional potential covariates such as medical comorbidities in the multivariate analysis); 2) findings from patients seeking care at a tertiary medical center because of the severity of their symptoms may not be generalizable to the general OAB population; and 3) assessment of depression was based on self-reported symptoms. Although the HADS-D is commonly used to screen for depression in the outpatient setting, it is not a diagnostic tool for clinical depression, and it cannot be ascertained without psychiatric evaluation which was not performed here. Despite these limitations, many of the comparisons clearly demonstrated differences. Large multi-institutional studies of the clinical OAB population are needed to further examine these relationships.

## Conclusions

About 30 % of OAB patients have depression. OAB patients with depression reported more severe urinary incontinence symptoms, greater bother and more impact on quality of life compared to those without depression. Future studies are needed to further examine the mechanistic links between depression and OAB/urinary incontinence.

## Appendix 1

**Table 5:** Description of the study population

	OAB patients	Controls	*p*-value (adjusted for age and sex)
Demographics:			
No. of subjects	51	30	
Age (mean ± SD)	53.8 ± 11.9	54.2 ± 12.3	0.98
Sex (% females)	73 %	57 %	0.14
Race (% white)	43.1 %	63.3 %	0.08
Age of diagnosis of OAB (mean ± SD)	47.5 ± 15.2	Not applicable	
% with OAB symptoms less than one year?	24 %	Not applicable	
Urinary symptoms: (mean ± SD)			
No. of daytime void (see footnote)^a^	1.8 ± 1.3	0.2 ± 0.6	<0.001
No of nighttime void^b^	2.6 ± 1.1	0.9 ± 0.7	<0.001
How often rush to bathroom to void?^c^	2.7 ± 0.9	0.6 ± 0.7	<0.001
How often does urine leak before getting to the bathroom?^d^	2.2 ± 0.9	0.3 ± 0.5	<0.001
Validated questionnaires: (mean ± SD)			
ICIQ-UI (urinary incontinence, 0–21)	12.0 ± 4.9	1.4 ± 2.0	<0.001
ICIQ-OAB (overactive bladder, 0–16)	9.3 ± 2.6	2.0 ± 1.5	<0.001
OAB-q symptom bother subscale (6–36)	19.1 ± 6.6	2.2 ± 2.8	<0.001
OAB-q quality of life subscale (13–78)	29.7 ± 16.9	2.0 ± 3.0	<0.001
UDI-6 (urogenital distress inventory, 0–24)	12.6 ± 5.6	0.9 ± 1.4	<0.001
IIQ-7 (incontinence impact questionnaire, 0–28)	8.8 ± 8.2	0.1 ± 0.4	<0.001
Medical comorbidities:			
Hypertension	37 %	33 %	0.72
Diabetes	8 %	3 %	0.31
Stroke, transient ischemic attack	8 %	7 %	0.15
MI, angina	0 %	0 %	1.00

Based on the categories of response on the ICIQ-OAB questionnaire

^a^How many times do you urinate during the day? 0 = 1 to 6 times, 1 = 7 to 8 times, 2 = 9 to 10 times, 3 = 11 to 12 times, 4 = 13 or more times

^b^During the night, how many times do you have to get up to urinate, on average? 0 = none, 1 = one time, 2 = two times, 3 = three times, 4 = four or more times

^c^Do you have to rush to the toilet to urinate? 0 = never, 1 = occasionally, 2 = sometimes, 3 = most of the time, 4 = all of the time

^d^Does urine leak before you can get to the toilet? 0 = never, 1 = occasionally, 2 = sometimes, 3 = most of the time, 4 = all of the time

## Abbreviations

HADS-D: Hospital anxiety and depression scale – depression subscale
ICIQ-OAB: International consultation on incontinence – overactive bladder
ICIQ-UI: International consultation on incontinence – urinary incontinence short form
IIQ-7: Incontinence impact questionnaire short form
OAB: Overactive bladder
UDI-6: Urogenital Distress Inventory Short Form
UI: Urinary incontinence
